# Synthesis and Properties of Sr_9_Ce_2_W_4_O_24_ Quaternary Perovskite

**DOI:** 10.1021/acs.inorgchem.5c04572

**Published:** 2026-02-18

**Authors:** Damian Wlodarczyk, Mikolaj Amilusik, Maciej Chrunik, Roman Minikayev, Paulina Kosmela, Michal Strankowski, Lev-Ivan Bulyk, Volodymyr Tsiumra, Marcin Zajac, Anastasiia Lysak, Sara Piotrowska, Michal Bockowski, Justyna Barzowska, Hanka Przybylinska, Andrzej Suchocki

**Affiliations:** † Institute of Physics, 86906 Polish Academy of Sciences, Aleja Lotnikow 32/46, Warsaw PL-02668, Poland; ‡ Institute of High Pressure, 243577 Polish Academy of Sciences, Sokolowska 29/37, Warsaw PL-01142, Poland; § 69698 Military University of Technology, Gen. Sylwestra Kaliskiego 2, Warsaw PL-00908, Poland; ∥ 431446 Gdansk University of Technology, G. Narutowicza 11/12, Gdansk PL-80233, Poland; ⊥ Solaris Synchrotron NSRC, 37799 Jagiellonian University, Czerwone Maki 98, Krakow PL-30392, Poland; # Institute of Experimental Physics, Faculty of Mathematics, Physics and Informatics, University of Gdansk, Wita Stwosza 57, Gdansk PL-80308, Poland

## Abstract

We report details on the synthesis and properties of strontium cerium tungstate, Sr_9_Ce_2_W_4_O_24_, a quaternary perovskite that has not yet been synthesized in pure form. Room-temperature powder X-ray diffraction (XRD) identified the most probable space group as tetragonal *I*4_1_/*a*. X-ray photoelectron spectroscopy confirmed that the material obtained after synthesis contained cerium in both 3+ and 4+ charge states, as well as tungsten in 5+ and 6+ states. Temperature-dependent Raman and XRD measurements done at 1 atm air and N_2_ excluded the possibility of a phase transition occurring within the 8–873 K; however, when exposed to air, the compound decomposes to a mixture of various salts and oxides just above 573 K. When compressed at room temperature, a possible transition toward lower-symmetry, monoclinic (*C*2/*c* or *C*2/*m*) space groups was detected between 12.5 and 15 GPa.

## Graphical abstract

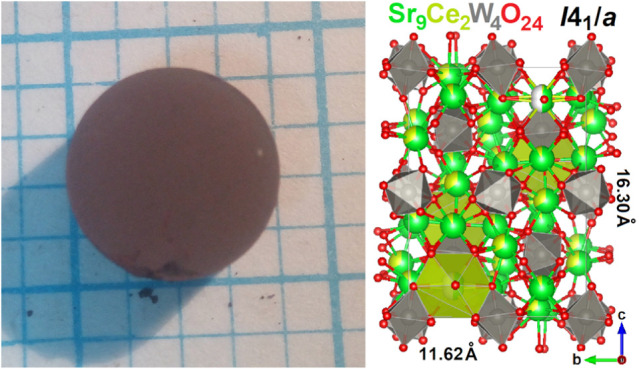

## 1 Introduction

When investigating and analyzing a variety of SrO-La_2_O_3_–WO_3_ quaternary oxides during the late 1980s, Smirnov^1^ first reported a system, an archetype of sorts, to our Sr_9_Ce_2_W_4_O_24_ (SCWO). Only after approximately 20 years, Xie^2^ and Zheng et al.^3^ developed and described similar structures denoted as Sr_9_RE_2_W_4_O_24_ (RE = Gd, Y), with their first CIFs and distinctive XRD patterns enlisted as JCPDS 50–0375 and 49–0568, respectively. The early 2010s brought to light Ba_9_Ln_2_W_4_O_24_ and a wide variety of doped, Sr-related 4 × 4 × 4 cubic (now-called quadruple) perovskites in the form of Sr_9_Ln_2_W_4_O_24_ (Ln = La, Pr, Nd, Sm, Eu, Gd, Mn, Y),^4–10^ but the accurate description of structural defects, their ordering, and PL capability came with the work of Fu^11^ and Ijdo et al.^8^— it was about Ba_2_M^(II)^M′^(VI)^O_6_ (M = Ca, Sr, M′=Te, W, U) and Sr_7/4_□_1/4_SrReO_6_ (where □ is denominated as a defect—vacancy). They both referenced Sr_11_Re_4_O_24_ & Ba_11_Os_4_O_24_ as their initial inspiration (based on ICSD 90927 entry).^12,13^ In recent years (2020–2025), these materials have been primarily tested as phosphors with anomalous orange-to-near-infrared emission (in the 550–1500 nm range) and as highly efficient quantum-cutting devices for potential applications, such as indoor plant cultivation.^14^ The most promising investigations concern the quantum efficiency of enhanced far-red-emitting luminescent materials exhibiting semiconductive behavior with thermal activation, as demonstrated by the works of Ijdo, Ye, and Han et al.^8–10^

As the description of this new, complex structure, as well as its stability and polymorphism, is the prime focus of this paper, we aim to contribute to the scientific discussion about the nature of this mineral group from a fundamental point of view—current debates direct their attention toward the identification of standard quaternary perovskite features, their stability, defect control, and photoluminescence-related effects. We agree that these materials form complex, 4a × 4a × 4a multilayered superstructures, where “a” denotes the primitive (single perovskite) unit cell dimension. Most of them, including our SCWO, crystallize as large, densely packed tetragonal structures containing 347 atoms per unit cell.^2–7,12–16^ Some can also adopt cubic (*Fm*-3*m*) SG;^3,4,7–10^ however, their common characteristic is a neat, long-range order of defects distributed across shared (RE^3/4+^/A^2+^) or partially occupied alkaline (A^2+^) sites. For the latter group, this cavity-ordering trait primarily occurs at anion oxygen sites surrounded by face-centered A^2+^ ions. Another distinctive feature is the modification of most^VI^B-site octahedra to tetrahedra, often shared by selected alkaline and RE atoms. These materials have also been reported to form spontaneously during synthesis when substituting one basic RE metal for another.^3,5,8,9^ We encountered a similar phenomenon while replacing large alkaline A^2+^ ions (such as Ba^2+^) with smaller atoms from the same group in the periodic table, like Sr^2+^ and Ca^2+^.^4,7^ While large barium ions formed classic Ba_2_(Ce/Pr)­WO_6,_ double perovskite (DP) unit cells (with Goldschmidt factor (GS) as high as ∼0.96)^17,18^ much smaller calcium ions produced a Ca_3_Ce_2_W_2_O_12_ ilmenite derivative (with much lower GS ∼0.89).^18^ Strontium, as an intermediate cation, generated less tilt and shrinkage at alkaline sites, resulting in a showcased, unique structure. Compared to ordered barium double perovskites, which have a total of 8+ charge among central (rare earth-transition metal) BB’-site ions, the newly created quaternary perovskite shifts the sum central pair charge value toward 9+ by introducing (□- a cavity) defects similar to those present in ilmenites. This nicely complements the smoothness of the gradually changing structure and physicochemical properties as tilt and disorder progress, whereas changes in A^2+^ ionic radii cannot compensate for the buildup of defects, as described by Vasala and Karpinnen in their review of double perovskites.^19^

## 2 Experimental Section

### 2.1 Characterization

Scanning Electron Microscopy (SEM) was performed using an ultrahigh-resolution, field-emission Hitachi SU-70 microscope equipped with a Thermo Scientific Ultra Dry Energy Dispersive X-ray (EDX) spectroscopy system, the Gatan MonoCL 3 CL spectrometer, SSD detector, and an HKL Channel5 EBDS setup. The distribution of crystallite sizes was analyzed using ImageJ. Powder X-ray diffraction (X’pert MPD, Panalytical) was recorded in Bragg–Brentano geometry with 0.05° resolution using the Cu K_α1_ line. FullProf Suite was used for Rietveld refinement. XPS measurements were performed on powders placed in a Prevac setup equipped with a Scienta R4000 200 eV hemispherical analyzer and a monochromatic Al K_α_ (∼1486.7 eV) X-ray tube. The spectra were analyzed using the CASA XPS software package, version 2.3.17. Shirley background correction was used, and peaks were fitted with mixed Gaussian–Lorentzian functions. Atomic content was determined with an accuracy of at least 0.5% for W and 1.1% for Ce. Powders were placed in quartz tubes. Raman spectra were recorded on an S&I GmbH MonovistaCRS+ spectrometer equipped with a 0.75 m Acton–Princeton monochromator, a nitrogen-cooled CCD camera with a back-thinned PyLoN system, an Olympus XYZ IX71 inverted stage microscope with a Moticam camera, a long working distance 50× objective (NA 0.9), and a diode-pumped Cobolt Samba 04–01 laser emitting at 532 nm. The spectral resolution was approximately 0.88 cm^1^. The LINKAM FTIR600 device was used to control the temperature in the 80–873 K range under an inert N_2_ atmosphere, incorporating a T96 controller (heater) coupled with the LNP96 (cooling) module. Samples (pellets) were usually placed on or sandwiched (powders) between thin quartz plates. Fourier Transform Infrared (FTIR) spectra were registered using a Nicolet 8700 spectrometer from ThermoElectron Corp. operating in the Attenuated Total Reflectance (ATR) mode. High-pressure (HP) measurements were performed up to 21 GPa in an Almax easyLab Diacell Cryo miniature diamond anvil cell (DAC) equipped with 450 mm culets. The pressure-transmitting medium was argon, and ruby was used as a pressure gauge. Gaskets with 0.15 mm diameter holes were fabricated from Inconel X750 alloy and filled with powder to approximatelly 50 vol %. All LT experiments below 80 K were performed using an Oxford Instruments continuous-flow helium cryostat (model CF1204). No uncommon hazards were noted.

### 2.2 Synthesis

The compound initially targeted by the project was Sr_2_CeWO_6_ (SCW) perovskite; however, after several attempts, it became evident that the natural balance of products obtained in the final batch was naturally shifting toward Sr_3_Ce_2_W_2_O_12_ (an ilmenite similar to already synthesized Ca_3_Ce_2_W_2_O_12_)^18^ and Sr_9_Ce_2_W_4_O_24_ (SCWO, considered a quaternary perovskite). Given the rarity and abundance of the latter, the reaction was optimized for SCWO, as shown in Figure 1.

**Figure 1:**
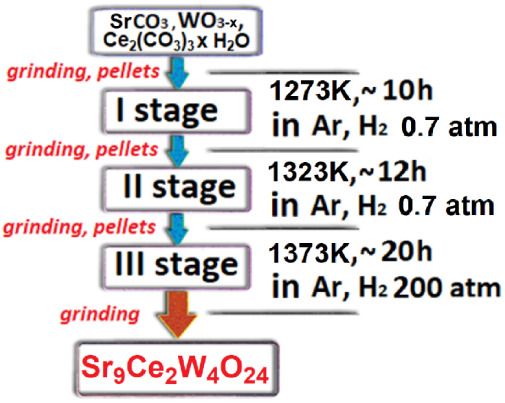
Sr_9_Ce_2_W_4_O_24_ solid-state reaction scheme with detailed information about each synthesis step.

The feasibility of Sr_9_Ce_2_W_4_O_24_ formation was checked by calculating the classic Goldschmidt^20^ (t) and the recently modified by Bartel^21^ (τ) tolerance factors for different pairs of BB′-site ions, as shown in Table 1. Although the parameters for both sets, Ce^4+^/W^5+^ and Ce^3+^/W^6+^, lie within the acceptable tolerance range, between 0.825 and 1.059, the former pair is closer to the ideal value of t = 1. However, we also wanted to create a PL-active compound that would function as an efficient down-converter. Therefore, an obvious choice was made to start with Ce^3+^ and W^6+^ oxides (Ce_2_O_3_ and WO_3_) as initial sources and compensate for emerging higher-valency ions as the solid-state reaction progresses. As for the latter, the WO_3_ substrate is stable and pure, whereas the former, Ce_2_O_3_, remains in its reduced state only briefly, even under reductive conditions. Given the slow oxidation of Ce_2_O_3_ to more prevalent CeO_2‑x_ species, we have chosen the more stable and accessible Ce_2_(CO_3_)_3_ hydrate as the substrate. Nevertheless, XRD detected 6% of the Ce^4+^ derivative after the substrate was dried. As the content of higher-valency Ce may still increase over time, we mitigated this by introducing Ar with a few parts per thousand of H_2_ during synthesis and by incorporating W_18_O_49_, which contains some W^5+^ ions, as a compensating agent. That reactant was obtained *“as is”* from the partial reduction of weighed WO_3_ using the Fita Chala^22^ method in order to maintain a total 9+ charge balance of B-site ions in SCWO and improve the overall GS factor.

**Table 1:** Classic Goldschmidt (t) and Modified (τ) Tolerance Factors Predicting the Existence of Sr_2_CeWO_6_ Quaternary Perovskite Depending on Ce and W Charge States^a^

Ionic radii [Å][Bibr ref70]				t	τ
^XII^A-site	^VI^B-site	^VI^B′-site	^II^X-site		
Sr^2+^ 1.44	Ce^4+^ 0.87	W^5+^ 0.62	O^2–^ 1.35	0.942	3.678
	Ce^3+^ 1.01	W^6+^ 0.60		0.916	3.829

a $t=\frac{rA+rX}{\surd 2\left(rBB^{\prime }+rX\right)};\;\tau =\frac{rX}{rBB^{\prime }}-2[2-\frac{rA}{rBB^{\prime }\times \ln\left(rA/rBB^{\prime }\right)};\;r_{I}-ionic\;radii;rBB’=1/2\left(rB+rB’\right)$

The initial substrates used for solid-state reaction synthesis were: WO_3_ (99.995%, Strem Chemicals), heated in a reducing atmosphere at ∼650 °C for 30 min (according to the aforementioned procedure). XRD measurements taken immediately afterward revealed that the intermediate product contained only 22.5% of the W_18_O_49_ phase (matching ICDD 05–0392 card) and two carbonates: SrCO_3_ (99.94%, Strem Chemicals) and Ce_2_(CO_3_)_3_ × n (6) H_2_O (declared 99% pure by Strem Chemicals, although XRD suggested 6% of Ce­(CO_3_)_2_)—both were preheated to remove the intercalar water. All three chemicals were weighed in the ratio 1.215:0.421:0.848 g (Table 2) to ensure proper stoichiometry of Sr, Ce, and W cations per 2 g of the final product and then mixed.

**Table 2:** Details about Substrates Used for Solid-State Reaction of SCWO, Remarks Regarding Their Stability, Pretreatment, and Content per Synthesized 2g Batch

Substrate	Purity [%]	Provider	Remarks	Content per 2g of final product [g]
SrCO_3_	99.94	Strem Chemicals	Preheated to release H_2_O	1.215
Ce_2_(CO_3_)_3_ × n (6) H_2_O	99^a^		Preheated to release H_2_O; 6% of Ce(CO_3_)_2_	0.421
WO_3_	99.995			0.848
WO_3‑x_ (W_18_O_49_)	22.5	Made by Fita-Chala method^22^	Unstable (desiccator in dark/max 2–3 days)	As acquired from the reduction of WO_3_

a Final purity of the substrate declared by the company was different than stated; The substrate was additionally dried and weighed.

Sr_9_Ce_2_W_4_O_24_ was synthesized by solid-state reaction in a mildly reducing mixture of 5.0-grade purity gases Ar:H_2_ (999–995:1–5 mL). This atmosphere was essential because any surplus of oxygen redirected the reaction toward other tungstate phases and prevented efficient bonding of the cerates. The synthesis took place in three stages. In the first stage, the pellet was heated to 1000 °C for 10 h in a constant-flow tube furnace at a gas pressure of 0.7 atm to release CO_2_. In the next stage, the temperature was increased to 1050 °C, while the incubation time was extended to 12 h. Gas pressure remained the same. In the final stage, the material was heated to 1100 °C for 20 h in a chamber pressurized to ∼200 atm to enhance ionic migration and fusion rates. Prior to each synthesis step, substrates were ground in an agate mortar and again hydraulically pressed into a pellet at approximately 10 MPa. Corundum (not graphite) crucibles were used to avoid carbon contamination and WC formation.

## 3 Results and Discussion

### 3.1 SEM Analysis

SEM images and photographs of the obtained material are shown in Figure 2. The hazelnut-brown SCWO pellet mainly consists of micrometer-sized, polygon-shaped grains embedded in fine submicron-to-nano powder. The average size of 20 randomly chosen grains selected from the image was about 1128.3 ± 104.9 nm (with a spread from 875 to 1313 nm). The Full Width at Half-Maximum (FWHM) of selectively chosen XRD peaks can give more comparative information on the particles’ size, according to the Debye–Scherrer equation,^23^ although the method is considered less accurate for grains larger than 200 nm. Compared with SEM imaging results, these estimates appear slightly underappreciated but still remain in good agreement. Based on shape constants (K ∼ 0.83 ÷ 0.86) for the three dominant peaks at 2Θ = 81.3, 72.8, and 44.1 and their FWHM’s, the determined average size is roughly 973 ± 43 nm. When heated in air, SCWO slowly decomposes into a beige-gray powder, starting from the surface (Figure S1). The oxidation mechanisms can vary but primarily involve the formation of the Sr_3_Ca_2_W_2_O_12_ phase, mixed with SrWO_4_ and various CeO_2‑x_ oxides.

**Figure 2:**
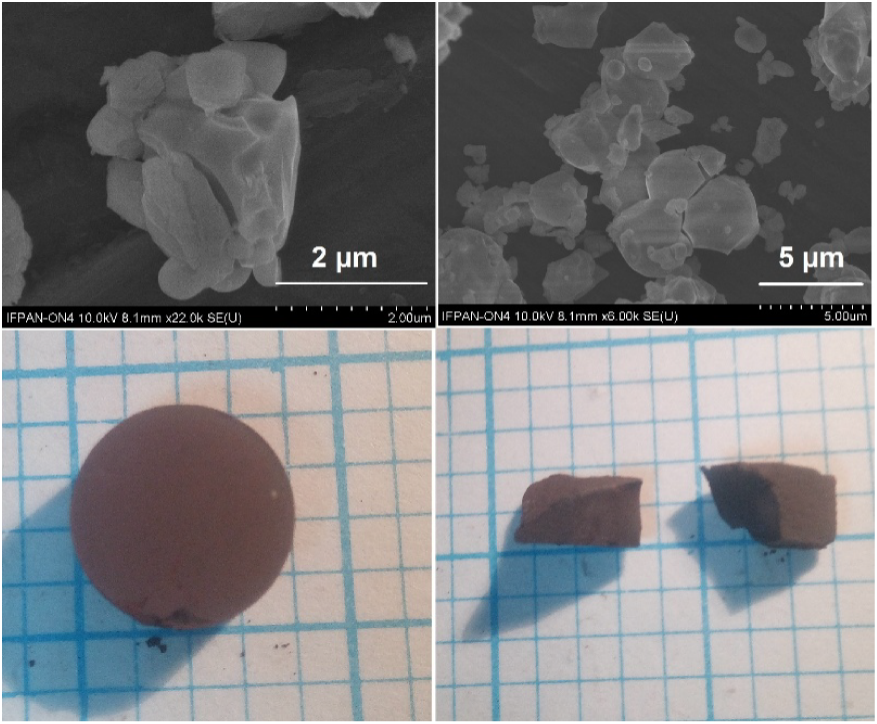
Upper panel: SEM micrographs of SCWO were obtained at two different magnifications. The scale is given in the graphs. Lower panel: photographs of the obtained pellets: whole (left) and cut-through (right).

### 3.2 Powder X-Ray Diffraction

The powder XRD pattern of the synthesized product, Sr_9_Ce_2_W_4_O_24_ (SCWO), is shown in Figure 3a and is isostructural with the one listed in the JCPDS 49–0568 card, which represents Sr_9_M_2_W_4_O_24_ (M = La and Y) compounds hosting the *I*4_1_/*a* space group. They are recognized as quaternary perovskites, often partially substituted with ions such as (Ho, Pr, Tm, Er, Bi, Nd, Sm, and Eu)^3+^ for PL modification purposes.^1–3,5,14,15^ The Rietveld refinement data fit the tetragonal pattern well, with no reproducible impurities detected in significant amounts (<1%). The main structure is shown in Figure 3b with the ascribed unit cell dimensions: *a*,*b* = 11.6220(9) Å, *c* = 16.2980(1) Å, and V = 2201.42(1) Å^3^. Other materials, like Ca_9_M_2_W_4_O_24_ (with M = Gd, Nd, Sm, Eu equivalent to the JCPDS 41–0186 pattern),^4,16,24^ or Ba_11_Os_4_O_24_, were also found to crystallize in a similar crystallographic group.^13^ All of them share the same quaternary oxide origin, first reported by Smirnov^1^ during the late 1980s, which was later described in greater detail by Bramnik et al.^12^ in the early 2000s, referring to Sr_11_Re_4_O_24_ as evidenced by the ICSD 90929 entry.

**Figure 3:**
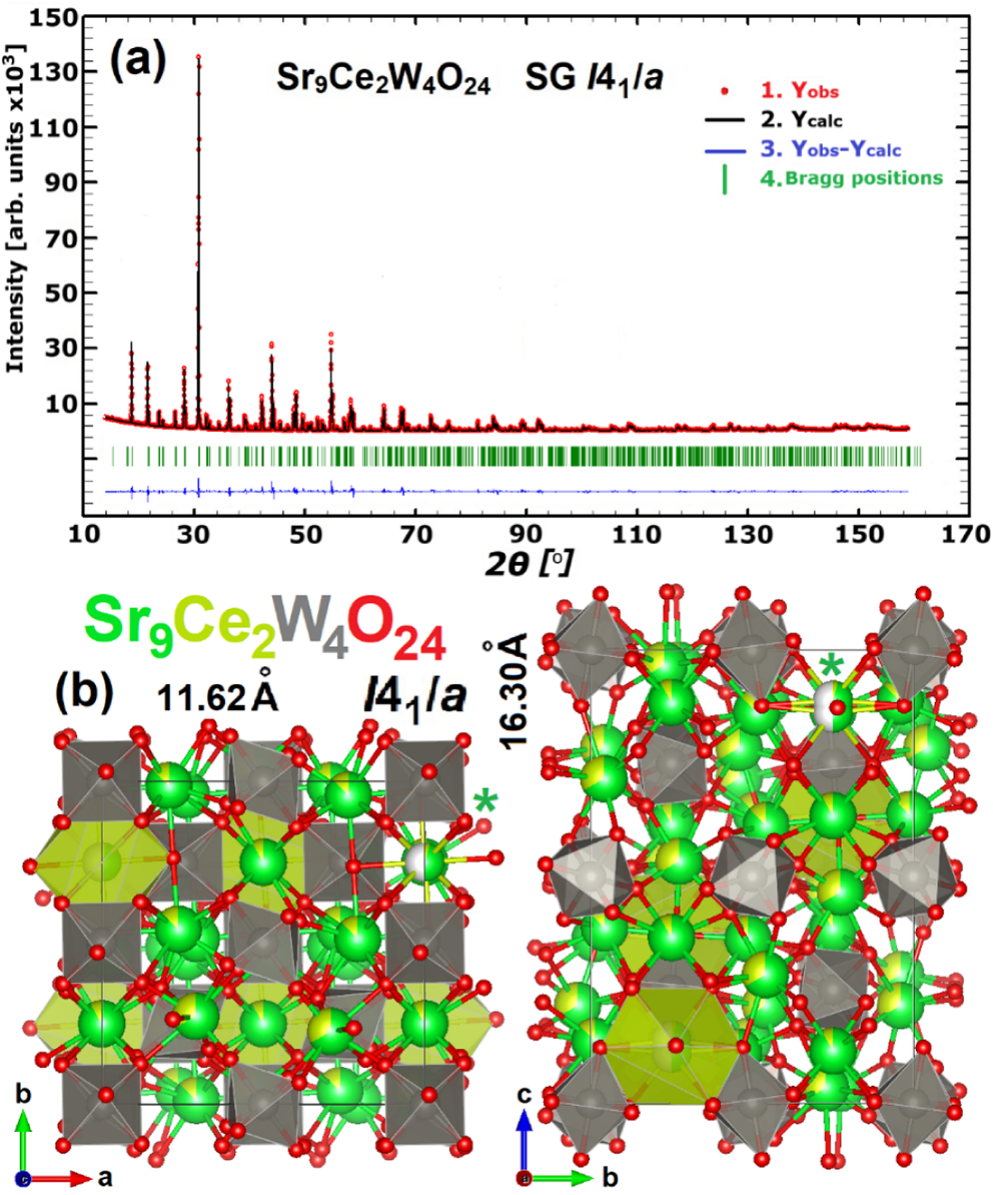
(a) XRD pattern of the final SSR synthesis product, Sr_9_Ce_2_W_4_O_24_. Dots represent experimental data; black line, Rietveld refined fit of the main SCWO phase to tetragonal *I*4_1_/*a* SG; blue line, difference between experimental and calculated data. Green bars denote Bragg positions for the three phases. Figure (b): projection of the unit cell on the *ab* (left) and *bc* plane (right). Green, yellow, gray, and red spheres denote Sr, Ce, W, and O ions, respectively.

There is some ambiguity regarding quaternary perovskites and their structural notations—particularly related to the cases of doped Sr_9_Gd_2_W_4_O_24_ and Ba_9_La_2_W_4_O_24_,^3,4,7–10,25^ where these densely packed compounds are riddled with uniformly dispersed vacancies (□). They usually host highly ordered, half-occupied oxygen defects localized at the corners and central faces of A^2+^ (here, Sr^2+^) dodecahedra, inspiring formula notations such as Sr_7/4_□_1/4_SrReO_6_.^8,11,16^ Adapting to this motif, our compound could also be written as [(Sr_5/4_□_1/4_C*e*_1/2_)­SrWO_6_]. The background-corrected Rietveld refinement parameters for the tetragonal SG are presented in Table 3, while the atomic coordinates and occupancy factors for the *I*4_1_/*a* SG are collected in Table 4.

**Table 3:** Rietveld Reliability Factors for the Diffractogram Presented in Figure 3a

Formula	SG	Z	V [Å^3^]	d_cal_ [g/cm^3^]	R_B_	R_P_	R_WP_	R_EXP_	N_σ_ GoF	χ2	Content [%]
Sr_9_Ce_2_W_4_O_24_	*I*4_1_/*a*	4	2201.421	6.602	3.81	11.1	13.2	6.24	223.05	4.51	100

**Table 4:** Atomic Coordinates and Occupancy Factors for All of the *I*4_1_/*a* Space Group Elements

Site label	x/*a*	y/*b*	z/*c*	occupancy
Sr1	0.20864(15)	0.22612(18)	0.53430(10)	0.643(5)
Ce1	0.20864(15)	0.22612(18)	0.53430(10)	0.357(5)
Sr2	0.2367(2)	0.04380(17)	0.11315(13)	0.873(7)
Ce2	0.2367(2)	0.04380(17)	0.11315(13)	0.127(3)
Sr3	0.00000	0.25000	0.36551(19)	0.972(9)
Ce3	0.00000	0.25000	0.36551(19)	0.028(1)
Sr4	0.00000	0.25000	0.1115(4)	0.497(1)
Ce4	0.00000	0.25000	0.1115(4)	0.003(9)
W1	0.00000	0.00000	0.50000	1.00000
W2	0.00000	0.00000	0.00000	1.00000
O1	0.0748(12)	0.0845(16)	0.5888(8)	1.00000
O2	0.1035(14)	0.0617(14)	0.4240(9)	1.00000
O3	0.0128(15)	0.0285(12)	0.1168(6)	1.00000
O4	0.1339(15)	0.1305(17)	0.2509(1)	1.00000
O5	0.3712(15)	0.1357(17)	0.2288(8)	1.00000
O6	0.3978(13)	0.1305(15)	0.0291(9)	1.00000

### 3.3 XPS and EDX Analysis

X-ray photoelectron spectroscopy (XPS) determines the standard atomic content of solids, including elements situated at various interstitial sites and residing in amorphous phases, which are usually not describable by powder XRD measurements. Although XPS can resolve multiple valence states of specific ions, its capability is somewhat limited, as measurements performed under ionizing radiation can alter the observed charge states at their apparent occupancies. Given the information above, we employ this technique solely to determine the analytical formula and provide basic data on the duality of a few crucial states in the final product.

The X-ray photoelectron spectrum of the Ce 3d_5/2_ and 3d_3/2_ spin–orbit split core levels (M_5_ and M_4_ edges, respectively) is shown in Figure 4a. The complex structure can be deconvoluted into three sets of doublets related to Ce^4+^, denoted as v, v″, v‴ in the d_5/2_ component and u, u″, u‴ in the d_3/2_ component, as green lines. The same was done for Ce^3+^ peaks, which are represented by two doublet sets denoted as vo, v′ (d_5/2_) and uo, u′ (d_3/2_), marked by blue lines. Following the peak notation and assignment of Romeo, Paparazzo, Nachimuthu, and others^26–34^ we established that most of the Ce ions are in the 3+ state, roughly 61%.

**Figure 4:**
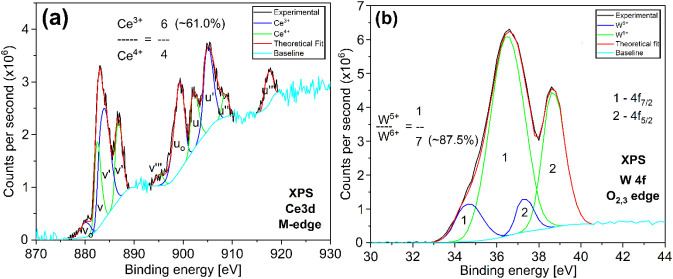
X-ray photoelectron spectra of the Ce 3d (a) and W 4f (b) core states. Red lines are fits to experimental data (black lines), taking into account two charge states for each ion (blue and green lines) and the background (cyan line).

In contrast to cerium, the XPS spectrum of tungsten 4f O_2,3_ edges shown in Figure 4b contains only two spin–orbit split doublets, denoted as “1” and “2” for the 4f_7/2_ and 4f_5/2_ core states, respectively. The peaks at binding energies (BE) of 34.7 eV (4f_7/2_) and 37.3 eV (4f_5/2_), depicted by the blue line, are assigned to W^5+^ and consist of 12.5% of the total surface area,^35–37^ while those at 36.5 and 38.5 eV (green line) are attributed to remaining W^6+^ ions.^38–40^ This indicates that the W^5+^ to W^6+^ ratio from the intermediate WO_3‑x_ substrate containing 22.5% W_18_O_49_ oxide remains relatively the same. Therefore, the previously reported elevated Ce^4+^ content, which should complement W^5+^ ions while maintaining the total 9+ charge balance at BB′-sites, must originate from sources other than the expected Ce→W charge transfer, possibly from residual oxygen or gases emitted from decomposing oxide substrates during synthesis.

The strontium 3d shell XPS spectrum is presented in Figure 5a. The two peaks related to the M_5_ edge 3d_5/2_ levels are assigned to signals positioned at (1) 131.2 &; (2) 133.0 eV, while the other pair related to the M_4_ edge 3d_3/2_ core states at (1′) 136 and (2′) 137.6 eV. The presence of 2 signals at each 3d shell closely reflects strontium’s charge states and indirectly describes the closest neighborhood. Tilting and shared occupancy (with Ce^3/4+^ ions) within the sample interfere with many classical Sr^2+^ sites (defined by a 22′ pair), by reducing their original charge state to 1+ and placing them in the minority.^41–43^ Most Sr dodecahedral sites are riddled with point defects and vacancies, hosting only single Sr­(+1)-O bonds—marked as (11′) bands.^44,45^ The surface area ratio of both sets is roughly (11′) 2:1 (22′). A similar division was seen in the Ca 2p_3/2_ and p_1/2_ shell XPS spectra of Ca_3_Ce_2_W_2_O_12_, where partial substitution between Ca and Ce ions was also noticed.^18^

**Figure 5:**
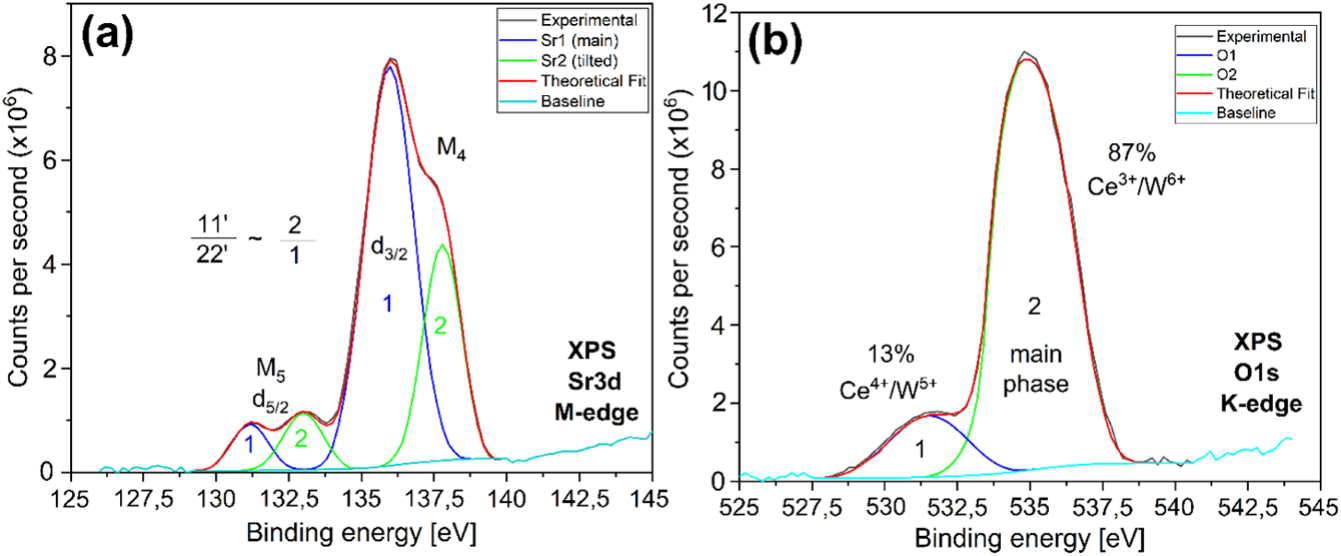
XPS spectra of the Sr 3d M_4,5_ shell (a) and the O 1s K-edge (b).

Partial substitution of alkaline ions could also be seen by analyzing the deconvoluted O 1s shell XPS spectrum in Figure 5b, where peaks at 531.0 and 534.6 eV are present (viewed as O^–^ and O^2–^ species, respectively).^46–48^ The peak’s area ratio suggests that they are rather quantitatively related to oxygen bonds connected to different BB′-site ion pairs (Ce^3/4+^ & W^5/6+^). The small peak, marked as 1, has a relatively small surface-area percentage (∼13%) that matches the amount of W^5+^ (∼12.5%). The higher-energy peak (no. 2) corresponds to the dominant W^6+^ ion. The analysis of the oxygen shell appears to be strongly correlated with the presence of defects and anions surrounding the imbalanced, inner BB′ states, which struggle to maintain the total charge state of 9+ (Ce^3+^/W^6+^, ΔZ = 3 vs Ce^4+^/W^5+^, ΔZ = 1).^49–52^

The positions of XPS peaks, their FWHM, and the determined percentage atomic content for all analyzed atomic species are collected in Table 5. From these data, it is evident that the analytical formula for the entire product is Sr_9.22_Ce_2_W_3.82_O_25.20_. This divergence from the initially planned formula, Sr_9_Ce_2_W_4_O_24_, as determined by XRD, is minimal. The oxygen content is slightly higher, likely due to interstitial oxygen trapped between sites during the use of basic oxygen substrates. This would also explain the partial oxidation of the Ce^3+^ ions. Tungsten might be slightly deficient because of its slow sublimation above 1100 °C—an effect already noted in our previous articles.^17,18^ It is worth noting that the actual charge states of the constituent ions cannot be precisely determined by our XPS experiments. The numbers given in Figures 4 and 5 pertain only to the relative charge distribution under ionizing radiation, which may affect the total ionic distribution within SCWO during prolonged exposure.

**Table 5:** Summary of XPS Investigations of Ionic Core Levels in SCWO: Peak Positions, FWHM, Percentage Atomic Contents, and Ratios (Normalized to Ce)

Core level	s-o split	Position [eV]	FWHM [eV]	Total content [%]	Fraction
W 4f	7/2	34.7; 36.5	1.47; 1.89	9.49	3.82
	5/2	37.3; 38.5	0.90; 1.25		
O 1s	–	531.0; 534.6	3.03; 3.01	62.63	25.20
Sr 3d	5/2	131.2; 133.0	1.45; 1.60	22.91	9.22
	3/2	136.0; 137.6	1.98; 1.55		
Ce 3d	5/2	880.4; 882.7; 885.1; 887.8; 896.4	2.28; 1.39; 2.52; 1.68; 1.80	4.97	2
	3/2	899.5; 901.6; 904.6; 907.8; 917.2	2.15; 1.72; 2.18; 1.62; 1.92		

Energy-dispersive X-ray spectroscopy (EDX) was additionally employed to verify the stoichiometry previously inferred from X-ray photoelectron spectroscopy (XPS). The characteristic EDX emission spectrum shown in Figure 6 was used to determine elemental atomic percentages (at%), summarized in Table 6. For consistency in the quantitative analysis, only the L-shell emission lines of heavier elements were considered, thereby reducing uncertainties associated with low signal-to-noise ratios and potential overlap of a few critical peaks. The reported oxygen content may be slightly underestimated due to inherent limitations of the technique, as light elements (Z < 11) are typically quantified with lower accuracy. The aluminum signal (labeled as 2) originates from the sample holder material. Based on the presented EDX data, the compound composition was estimated to be approximately Sr_8.55_Ce_2_W_3.88_O_23.38_. This composition is in close agreement with the stoichiometry obtained from XPS (Sr_9.22_Ce_2_W_3.82_O_25.20_) and with the idealized formula Sr_9_Ce_2_W_4_O_24_ derived from X-ray diffraction (XRD) analysis.

**Figure 6:**
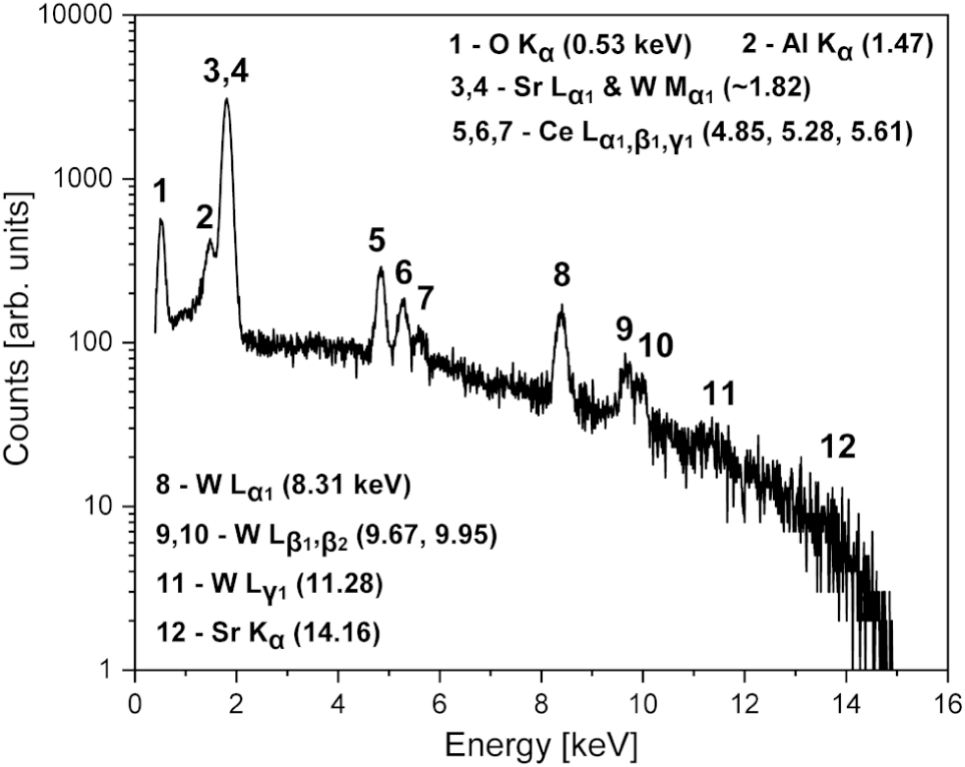
EDX spectrum of SCWO powder placed in an aluminum sample holder under 15 keV beam excitation.

**Table 6:** Quantitative Analysis of Chosen, Relevant SCWO Peaks from the EDX Spectrum Presented in Figure 6

Element line	Net Counts	Weight [%]	Atom [%]	Ratio
Sr L	35947	35.39	22.61	8.55
Ce L	6001	13.23	5.29	2
W L	2781	33.71	10.26	3.88
O K	6252	17.67	61.84	23.38
Total	50981	100	100	

### 3.4 Raman and FTIR Spectroscopies

Raman and FTIR spectroscopy were employed to establish the characteristic vibrational fingerprints of SCWO, providing a robust reference framework for future investigations of polymorphism and the stability of its tetragonal phase under nonambient conditions. Room-temperature Raman and FTIR spectra are compared in Figure 7, while deconvoluted peak positions and mode assignments, based on the literature^17,18,53–59,74–77^ are summarized in Table 7.

**Figure 7:**
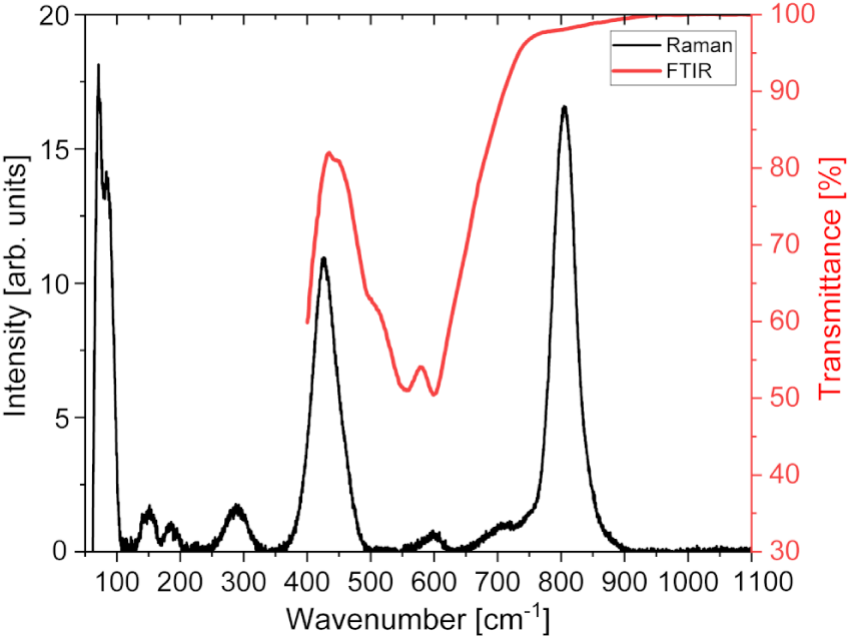
Raman (black line and axes) and FTIR (red line and axes) spectra of SCWO recorded at ambient conditions. No signals were detected above 1100 cm^–1^ (up to 4000 cm^–1^).

**Table 7:** Experimental Raman and FTIR Data Obtained at Ambient Conditions, with Literature-Based Phonon assignments^a^ ^17,18,53–59,74–77^

**Raman modes**		**IR modes**	
ω_0_ (cm^–1^)	assignment	ω_0_ (cm^–1^)	assignment
71.1	A-site lattice translations of mixed B_g_ and ^1^E_g_/^2^E_g_ origin	445	in- and out-of-plane bending MO_6_ bands of ^1^E_u_/^2^E_u_ origin down to 100 cm^–1^
85.0		497	ν_sym_ stretch of MO_6_ octahedra of A_u_ origin
141.5		554	
150.9		599	
186.5	in-plane σ_ρ_ and out-of-plane σ_τ_ bending in MO_6_ octahedra of mixed B_g_ and ^1^E_g_/^2^E_g_ origin.	648.5	ν_asym_ stretch of MO_6_ octahedra of A_u_ origin
289.3		797	
391.3		874	
411.4	in-plane σ_sc_ and out-of-plane σ_ω_ bending in MO_6_ of mixed B_g_ and ^1^E_g_ origin		
426.0			
557.6	transverse MO_6_ motion of mixed A_g_ character/possible CeO_2‑x_ impurities and (Ce^3+^:Ce^4+^)O_6_ V_o_ lattice distortions		
598.7			
710.9			
749.2	v_asym_ and v_sym_ oxygen stretches of A_g_ symmetry for various Wyckoff sites not only partially occupied but also hosting differently charged W ions		
784.7			
805.6			
844.2			
			

a v_sym_ – symmetric stretching; v_asym_ – asymmetric stretching; σ_sc_ – scissoring deformation: σ_ω_ – wagging deformation; σ_τ_ – twisting deformation; σ_ρ_ – rocking deformation; M – Ce or W ions.

The FTIR spectrum is included only as complementary support, as most low-frequency modes below ∼400 cm^–1^ are inaccessible due to instrumental limitations associated with nitrogen purging. As shown, the number of experimentally resolved Raman modes (16) is lower than theoretically predicted (48) for the tetragonal *I*4_1_/*a* space group (11A_g_ + 11B_g_ + 13^1^E_g_ + 13^2^E_g_; see Table S1 in the Supporting Information for reference). This discrepancy is attributed to mode degeneracy and overlap arising from mixed Ce^3+^/Ce^4+^ and W^5+^/W^6+^ occupancies at the B sites, as well as partial Sr/Ce site sharing.

Consistent with this interpretation, the FTIR features are significantly broader (FWHM ≈ 48–58 cm^– 1^) than their Raman counterparts (21–36 cm^–1^), reflecting the combined effects of shared occupancies, point defects, and enhanced local disorder, which limit the experimental resolution and significance of individual phonon modes’ interpretation. However, a particularly informative comparison can be drawn with the work of Ratheesh et al.,^60^ who investigated structurally analogous rare-earth perovskites in which W is replaced by Ta, namely Sr­(B­(RE)_0.5_/(Ta or Nb)_0.5_)­O_3_ (B = Ga, In, Y, La, Pr, Nd, Sm, Eu, Gd, Tb, Dy, Yb), in the context of microwave integrated circuit applications. Their Raman spectra exhibit strong similarities to these observed here, including mode grouping, frequency evolution, and chemical mode assignments. Consistent with numerous prior studies, they demonstrate that Raman spectroscopy is sensitive enough to probe ordering and tilting of A- and B-site dodecahedral and octahedral sites by monitoring sharpness, intensity, and splitting of characteristic modes. We emphasize that crystal structure determination cannot rely solely on Raman mode splitting. However, when interpreted in conjunction with XRD analysis and the broad Raman literature on structurally related perovskites, the observed spectral features can be consistently rationalized in terms of octahedral tilting, local distortion, and partial cation disorder. Compared with these complementary observations, the resulting framework is coherent and robust, substantially strengthening the discussion below.

In particular, systematic changes in the spectral regions 50–200, 350–450, and 700–850 cm^–1^ are usually accompanied by symmetry-lowering features while transitioning from cubic (most common perovskite space group) to tetragonal^61^ (our case) or rhombohedral^62^ phases as the Goldschmidt factor decreases.^19,20^ During this process, increased peak broadening is generally associated with growing structural disorder, especially in compounds containing multiple rare-earth cations with nonfixed crystallographic positions. It is well established that decreasing symmetry (of a cubic phase) correlates in many ways with enhanced broadening of both the A_1g_ and F_2g_ modes. The lowest-frequency mode (∼70–100 cm^–1^), commonly assigned as F_2g_ modes in cubic perovskites, frequently splits into (in our case) two or more (4) components (i.e., B_g_) upon transitioning to low-symmetry phases, serving as a key indicator of reduced long-range order, octahedral tilting, or the presence of an entirely different phase. Even when full splitting is not completely resolved, the appearance of shoulders in this region is widely regarded as evidence of symmetry lowering (cubic → tetragonal/rhombohedral phase) and progressing cation disorder.^63–68^

The small difference between the ionic radii of Sr^XII^ (≈1.44 Å) and Ce^3+,XII^ (≈1.34 Å) makes partial A-site intermixing plausible.^70^ In that case, low-frequency features must be considered together with changes observed in the highest-frequency A_1g_ modes near ∼800 cm^–1^, which are responsible for B­(Ce)/B′(W)­O_6_ stretching modes; their intensity and asymmetry features are likewise sensitive to tolerance factor variations but not as much as low-frequency modes. Previous studies^60–69^ report that compounds with 0.92 < *t* < 0.90 typically exhibit broadened, asymmetric A_1g_ modes, whereas more symmetric F_2g_ behavior is observed for 0.94 < *t* < 0.92. Given the coexistence of Ce^3+^/Ce^4+^ and W^5+^/W^6+^ in SCWO, the effective tolerance factor is expected to span a relatively broad range (∼0.916–0.942), encompassing both regimes. The simultaneous presence of these spectral features is therefore anticipated and is consistent with observations reported by Trunov and Takahashi et al. for (La,Pr)-containing B′-site perovskites.^61,62^

These last-mentioned systems, which crystallize in the same tetragonal *I*4_1_/*a* space group as our SCWO, exhibit likewise incomplete resolution of low-wavenumber modes (now commonly assigned as B_g_) due to pronounced broadening (with shoulders stretching toward higher wavenumbers), as well as asymmetric features at the base of the A_1g_ band (stretching down to lower wavenumbers). The Raman spectra of Sr-, Pr- and La-based tantalates are particularly similar in overall shape to that of our compound. Notably, SCWO possesses substantially larger linewidths (by +8–10 cm^–1^ as FWHM of the Raman mode is ∼21–36 cm^–1^), suggesting an enhanced degree of local disorder, likely arising from the additional variability introduced by mixed Ce^3+^/Ce^4+^ and W^5+^/W^6+^, which was unfortunately omitted in the work of Ratheesh, Trunov & Takahashi et al.^60–62^ Interestingly, the E_g_ modes localized in the 450–550 cm^–1^ range remain comparatively insensitive and consistent in their characteristics, which indicates that we are surely not dealing with a trigonal phase.^71,72^ Their assignment has, to date, been associated solely with oxygen motions within the context of B-site distortions, or rather defect-related displacements, without any other meaningful features.^17,18,60,73^

Raman studies were performed at nonambient conditions to search for possible phase transitions. These usually follow a specific path, a sequence determined by the initially chosen space group, as suggested by Howard et al.^19,78,79^ Raman spectra recorded at low temperatures (LT) are presented in Figure 8a, and the temperature dependence of the peak positions is shown in Figure 8b. There are no discernible signs of change within the presented temperature range (while heating from 8 K to RT). All of the presented peaks shift linearly, albeit slightly, as they gradually broaden and merge. There are, however, three additional modes (marked with asterisks in Table 8) relative to the original RT spectrum previously mentioned in Figure 7. They are prevalent throughout the entire LT experiment and, according to the literature, result from another local (Ce^3+^:Ce^4+^)­O_6_ deformation and from apparent CeO_2–x_ impurities.^80–83^

**Figure 8:**
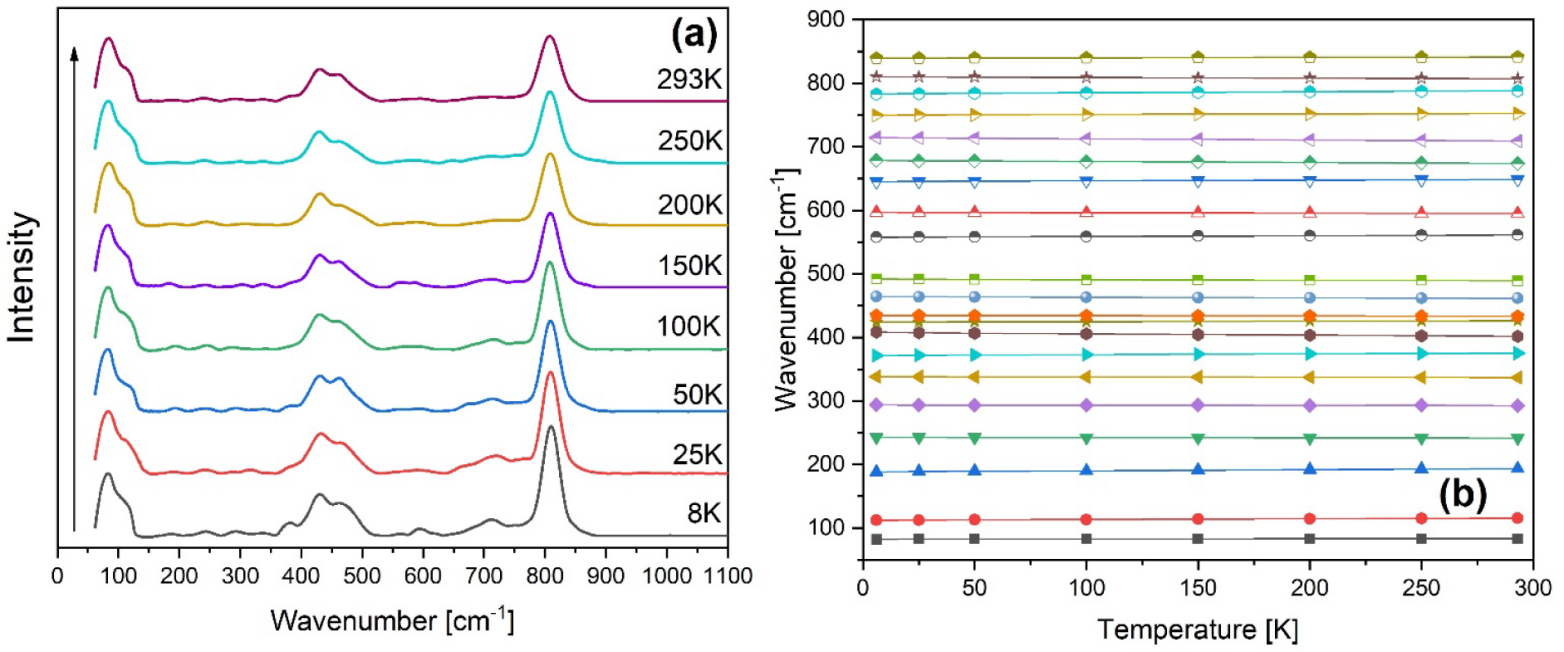
(a) Temperature-dependent Raman spectra under cryogenic conditions. (b) The changing Raman peak positions (symbols) shown with linear fits (solid lines).

**Table 8:** Raman Modes at 293 K (ω_RT_), and Temperature Coefficients (dω_LT_/dT) alongside RT Peak Positions in Ar atm^a^

ω_RT_ (cm^–1^)	dω_LT_/dT (cm^–1^/K)	ω_P_ (cm^–1^)	dω_P_/dP (cm^–1^/GPa)
83	0.0031 ± 0.0002	80	– 0.25 ± 0.01
115	0.0113 ± 0.0005	112	– 1.06 ± 0.04
193	0.0174 ± 0.0005	190	1.70 ± 0.05
242	– 0.0041 ± 0.0001	263	0.19 ± 0.03
293	– 0.0027 ± 0.0006		
337	– 0.0047 ± 0.0001	321	3.79 ± 0.11
375	0.0121 ± 0.0005		
402	– 0.0218 ± 0.0007	400	1.01 ± 0.05
426	0.0064 ± 0.0001	430	1.67 ± 0.04
434	– 0.0041 ± 0.0001		
461^b^	– 0.0099 ± 0.0004	462^b^	2.53 ± 0.09
489	– 0.0100 ± 0.0008		
561^b^	0.0120 ± 0.0003	558^b^	1.47
595^c^ ^b^	– 0.0066 ± 0.0002	593^c^ ^b^	1.95 ± 0.10
649^c^	0.0116 ± 0.0003	615^c^	2.51 ± 0.08
674^c^	– 0.0156 ± 0.0004	670^c^	4.31 ± 0.18
709	– 0.0189 ± 0.0002	695	3.12 ± 0.05
		714	4.70 ± 0.09
752	0.0090 ± 0.0005	754	3.88 ± 0.07
788	0.0172 ± 0.0005	788	4.07 ± 0.09
807	– 0.0100 ± 0.0003	808	4.00 ± 0.07
841	0.0067 ± 0.0002	852	3.37 ± 0.11
		1041^b^	– 0.18 ± 0.07

a (ω_P_) and their pressure coefficients (dω_P_/dP) up to 12.5 GPa.

b - Peaks originating from local CeO_2‑x_ impurities (#).

c - Unstable peaks influenced by Ce^3/4^O_6_ distortions (*).

RT Raman spectra under compression and decompression cycles are presented in Figure 9a,b and c, respectively, while the peak positions vs increasing and decreasing pressure are depicted in Figure 9d,e. Typical linear blue-shifting Raman modes are observed with increasing pressures up to 12.5 GPa, and their pressure coefficients are given in Table 8. Some extra signals were again observed and are marked with (b,c) annotations in tables, respective to asterisks and hashtags in the figures. Their origin is the same as previously described at the LT measurements. Some peaks are so weak that their pressure dependence cannot be followed in the whole range—this mainly concerns debatable modes related to CeO_2–x_ defects.^80–83^ Phase transitions at elevated pressures cannot be ruled out, as the material exhibits markedly nonlinear behavior above ∼15 GPa. Specifically, four additional Raman modes emerge above 500 cm^–1^, while the remaining modes undergo significant broadening, deviating from linear pressure dependence, and exhibit pronounced hysteresis upon decompression of approximately 3.5 GPa. These observations may be tentatively associated with a reduction in structural symmetry— consistent with the *I*4_1_/*a* → *C*2/*c* transition proposed by Howard et al.^78,79^ —is potentially driven by enhanced octahedral tilting or the onset of partial amorphization. However, contributions from deviatoric stress effects, arising from the progressively nonhydrostatic behavior of the Ar pressure-transmitting medium above ∼10 GPa in the diamond anvil cell, cannot be excluded either.^84^

**Figure 9:**
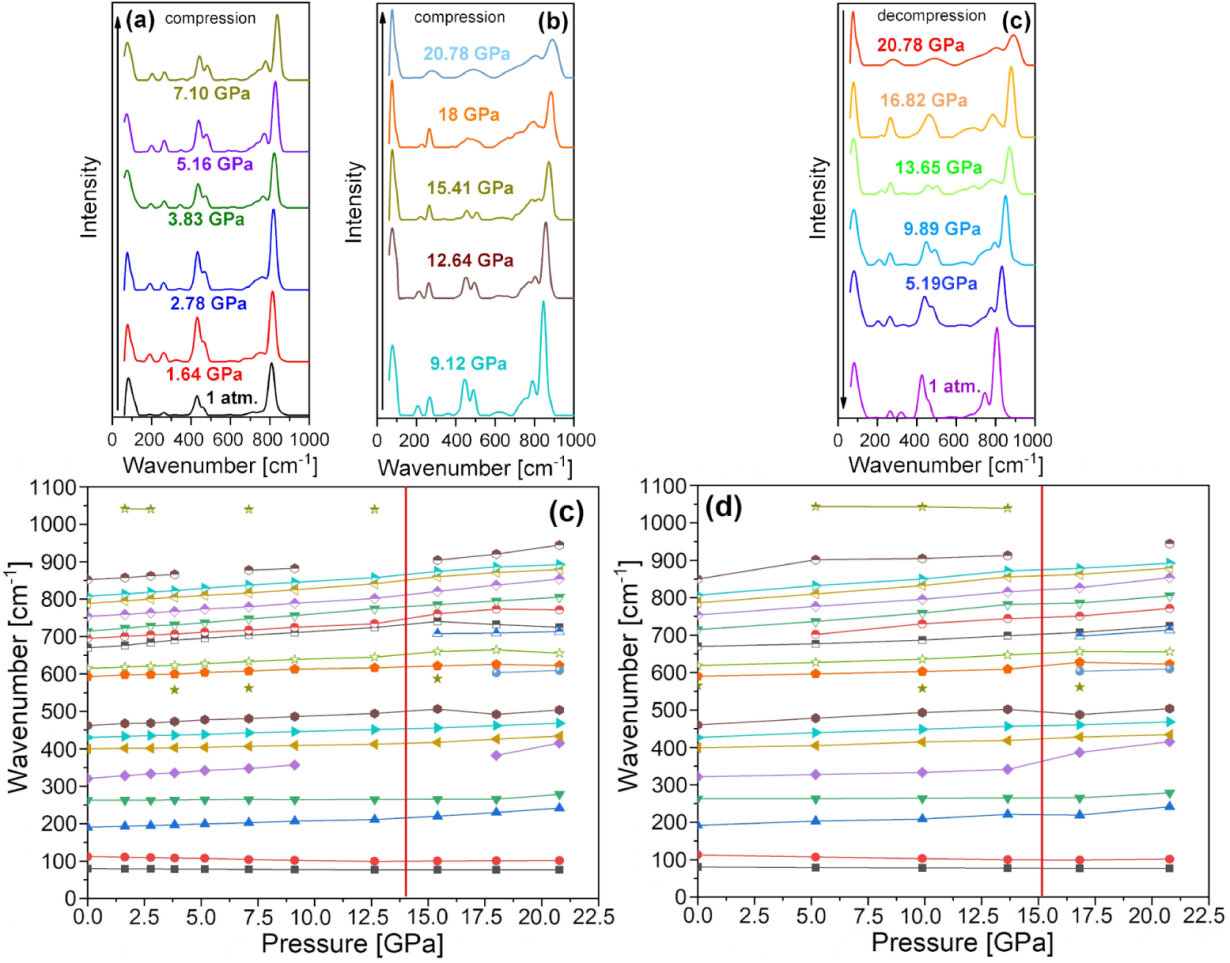
High-pressure Raman spectra (normalized to the peak at ∼82 cm^–1^) collected consecutively under compression (a,b) and decompression (c) cycles. Peak positions (symbols) versus increasing (d) and decreasing (e) pressure are depicted with the respective solid lines guiding the eye. Linear fits to the data presented in Table 8 were performed solely for the compression cycle from ambient pressure up to the points cut off by the solid, red, vertical lines, which signify the zone before any signs of a possible phase transition occur (between 12.5 and 15 GPa).

### 3.5 Material Stability at High Temperatures

The stability of SCWO at high temperatures is essential for potential applications, *e.g.,* as a down-converter in solar panels. Therefore, X-ray diffraction and Raman studies were performed up to 600 °C (873 K), in both air and inert N_2_. DSC and TG were also performed within a similar temperature range to corroborate the observed claims, given the importance of the heat capacity values and the energetic effects of potential transitions that may occur during heating. The latter two techniques are discussed using all collected data in Figure S2 of the supplementary file.

Temperature-dependent Raman and XRD spectra of SCWO are presented in Figure 10. When heated in a protective N_2_ atmosphere (Figure 10a), some minor changes in the spectra were observed above 573 K. Along with a barely noticeable linear red shift, some peaks’ intensity (marked with #) increased disproportionally in comparison to the other signals—their previous assignment, in Table 7, was associated with modes originating from mixed-valence, distorted Ce^3/4+^O_6_ octahedra. In air, the situation is much more complex. In Figure 10b,c, the Raman spectra and XRD patterns change considerably above 473 K. In the Raman spectra, modes related to Sr_*x*_WO_3+x_ and CeO_2‑x_ species (marked respectively by asterisks and hashtags in Figure 10b) gradually appear,^80–83,85–87^ indicating pronounced decomposition of SCWO. The original XRD pattern broadens and becomes weaker, while new sharp lines, related mainly to tungstates and distorted cerium oxides, appear and gain intensity. Below that threshold, SCWO’s reflections only shift and broaden slowly with increasing temperature, indicating gradual lattice expansion and amorphization without any structural transitions.

**Figure 10:**
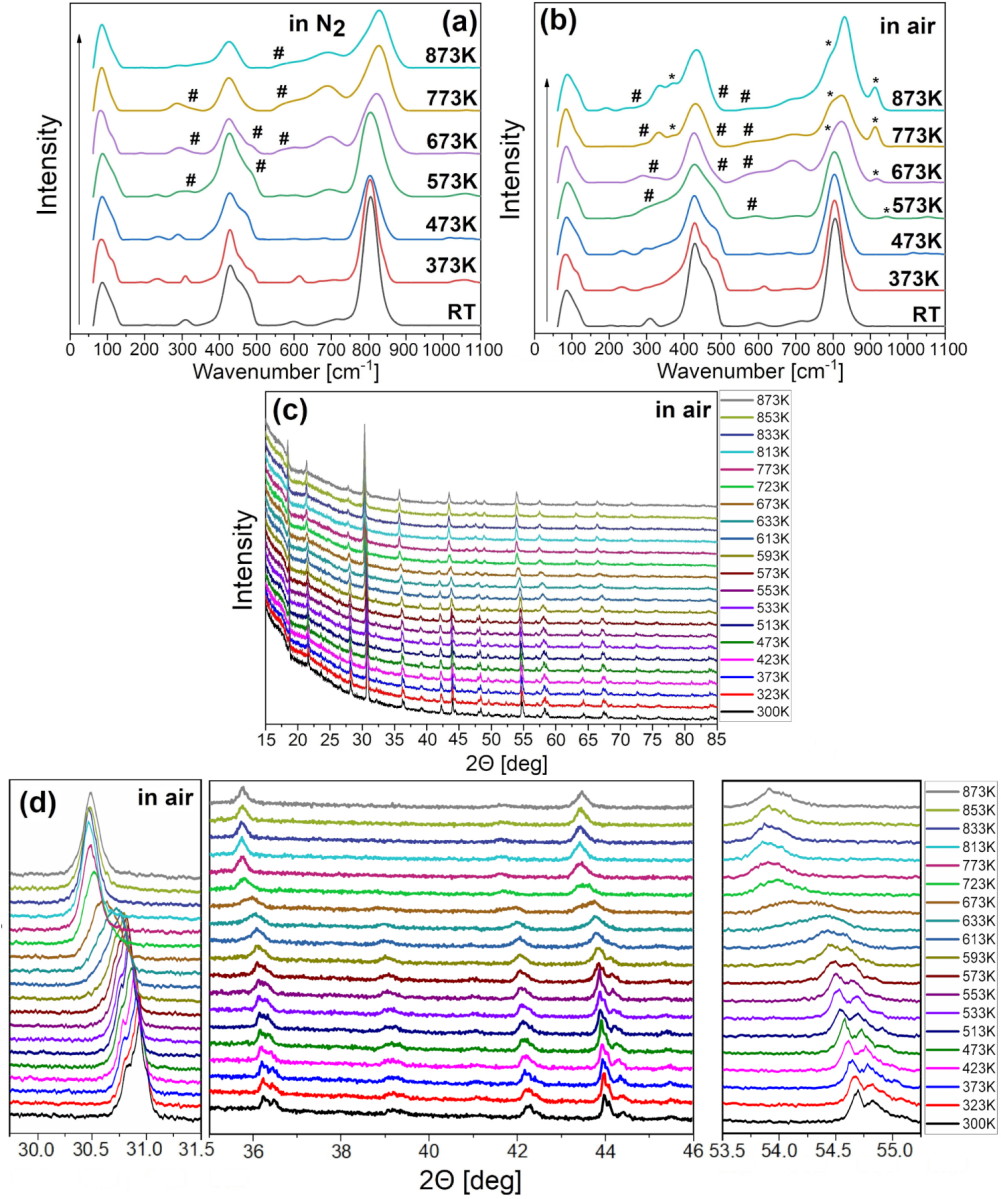
Temperature-dependent Raman spectra in inert N_2_ (a) and air atmospheres (b). Complementary powder XRD patterns recorded in air (c) confirm the onset of thermal decomposition at elevated temperatures, as shown with greater detail in enlarged regions of interest in graph (d).

The temperature dependencies of the unit cell parameters, determined from XRD measurements (performed in air) within the stable 300–550 K temperature range, where no decomposition signs were noted, are shown in Figure 11. The determined temperature coefficients for the “*a = b”,* and “*c”* lattice constants are 12.81(7) ± 0.35(0) × 10^–5^, and 29.01(7) ± 0.48(7) × 10^–5^ Å/K, respectively. The unit volume expands at a rate of 8.78(3) ± 0.0019(7) × 10^–2^ Å^3^/K while α, β, and γ angles remain close to 90°, confirming the lasting presence of tetragonal symmetry.

**Figure 11:**
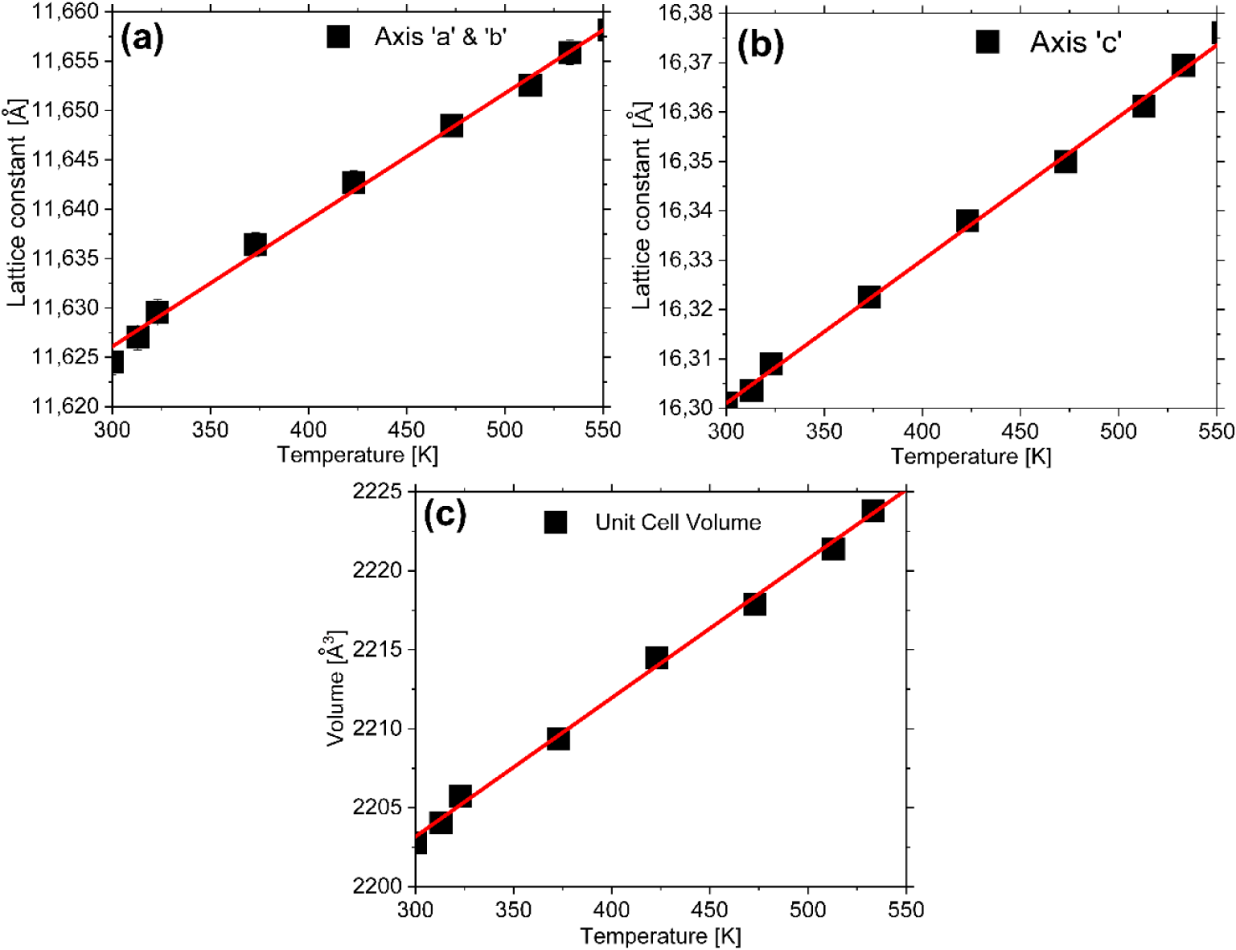
Temperature dependencies of lattice parameters: (a) *a* = *b*, (b) *c*, and (c) the whole volume of the unit cell. Solid lines are linear fits.

## 4 Summary

The newly synthesized strontium cerium-tungstate quaternary perovskite, Sr_9_Ce_2_W_4_O_24_ (SCWO), is reported. XRD studies have shown that the crystal structure of SCWO is substantially pure and possesses a tetragonal space group of *I*4_1_/*a* with *a*,*b* = 11.6220(9) Å, *c* = 16.2980(1) Å, and V = 2201.42(0) Å^3^ (R_wp_ ∼ 13.2%). CIF structural data are provided in Supporting Table S2. XPS investigations have shown that the freshly acquired material contains both (dominant) Ce^3+^ and (minority) Ce^4+^ ions as well as (minority) W^5+^ and (dominant) W^6+^. The composition derived from this technique unambiguously indicates an effective stoichiometry closer to Sr_9.22_Ce_2_W_3.82_O_25.2_, while EDX analysis suggests Sr_8.55_Ce_2_W_3.88_O_23.38_, reflecting the known limitations of EDX light elements’(oxygen) quantification.

The reduced number of Raman-active modes observed at room temperature deviates from group-theoretical predictions for ideal tetragonal symmetry, likely due to mode degeneracy arising from partial Sr/Ce site occupancy and mixed Ce^3+^/Ce^4+^ and W^5+^/W^6+^ valence states. This interpretation is further supported by the pronounced line-width broadening of several modes, indicative of enhanced structural disorder associated with a high density of point defects, which is characteristic of complex quaternary perovskites.

Above ∼15 GPa, an increase in the number of Raman-active modes is observed, which may indicate a transition to a lower-symmetry phase, tentatively assigned to *C*2/*c* or *C*2/*m* based on group-theoretical considerations by Howard et al.^78,79^ However, contributions from deviatoric stress associated with the progressively nonhydrostatic behavior of the Ar pressure-transmitting medium above ∼10 GPa cannot be excluded. Consequently, the observed spectral changes may also reflect stress-induced disorder or partial amorphization. Definitive discrimination between these scenarios will require complementary high-pressure XRD measurements and Raman experiments employing a more hydrostatic medium, such as helium. With an increase in temperature, many modes also broaden considerably and can no longer be detected, suggesting acute amorphization. These claims could be confirmed by HT XRD measurements, which reveal rapid, uneven lattice expansion, as evidenced by the coefficients. Raman and XRD spectra also indicate that SCWO is relatively stable up to at least 870 K under inert conditions, but it oxidizes rapidly when heated in air above 550 K, decomposing to SrWO_4_ and CeO_2‑x_. This was additionally confirmed by DSC and TGA measurements. We emphasize that, in the context of phosphor materials, the achieved air stability at 500–600 K is not exceptional and should be regarded as a baseline requirement. The presented fundamental study indicates that SCWO is unsuitable for high-temperature processing or prolonged operation in oxidative environments without inert gas protection, encapsulation, or use of protective coatings, particularly under repeated thermal cycling, because it is essentially a very good absorber. Such limitations would likely manifest during PL experimentation as rapid lumen depreciation and color instability in phosphor-based devices (especially in energy-conversion devices). While typical LED operating temperatures are lower (120–200 °C), local thermal hot spots could still induce local, irreversible degradation. Consequently, the applicability of SCWO in display technologies and high-temperature optical thermometry is restricted, though not entirely excluded, even under controlled conditions. The material remains viable for low-temperature (≤300 °C) NUV- or deeper-radiation sensing without requiring an inert or encapsulated environment. It also offers some value as a model system for fundamental studies of thermally activated processes, defect chemistry, and “structure–property” relationships.

## Supplementary Material



## Notes

DSC and TGA results (PDF)

## ∇

Late Prof. Andrzej Suchocki: 18th November 2025.

## Abbreviations

UV: ultraviolet
DPs: double perovskites
TM: transition metals
RE: rare-earth (ions or atoms)
SCWO: Sr_9_Ce_2_W_4_O_24_
CCWO: Ca_3_Ce_2_W_2_O_12_
BCW: Ba_2_CeWO_6_ double perovskite
BPW: Ba_2_PrWO_6_ double perovskite
XRD: X-ray diffraction
XPS: X-ray photoelectron spectroscopy
SG: space group
FTIR: Fourier Transform Infrared (spectroscopy)
SEM: Scanning Electron Microscopy
EDX: Energy dispersive X-ray radiation detector
FWHM: Full Width at Half Maximum
CCD: Charge Coupled Device
NA: numerical aperture
RT: room temperature
PLE: photoluminescence excitation
PL: photoluminescence
NIR: near-infrared, LT – low temperature
HP: high pressure
DAC: Diamond Anvil Cell
SSR: Solid State Reaction
GS: Goldschmidt factor
mGS: modernized Goldschmidt factor
ICSD: Inorganic Crystal Structure Database
CCDC: Cambridge Crystallographic Data Center
JCPDS: Joint Committee on Powder Diffraction Standards
BE: Binding Energy
O_i_: interstitial oxygen
NUV: near-ultraviolet
HT: high temperature
DSC: Differential Scanning Calorimetry
TG: Thermogravimetry
