# Supplementary material for: Synthesis and Properties of Sr9Ce2W4O24 Quaternary Perovskite
Source: Inorg Chem. 2026 Feb 18;65(8):4398–411. doi: 10.1021/acs.inorgchem.5c04572 (PMC13298893; doi:10.1021/acs.inorgchem.5c04572)
Supplement: Supplementary file 1 [file ic5c04572_si_001.pdf]

Supporting Information

Synthesis and properties of $Sr_9Ce_2W_4O_{24}$ quaternary perovskite

**Damian Wlodarczyk[1], Mikolaj Amilusik[2], Maciej Chrunik[3], Roman Minikayev[1], Paulina Kosmela[4], Michal Strankowski[4], Lev-Ivan Bulyk[1], Volodymyr Tsiumra[1], Marcin Zajac[5], Anastasiia Lysak[1], Sara Piotrowska[1], Michal Bockowski[2], Justyna Barzowska[6], Hanka Przybylinska[1], Andrzej Suchocki[1†]*

[1]Institute of Physics, Polish Academy of Sciences, Aleja Lotnikow 32/46, PL-02668, Warsaw, Poland

[2]Institute of High Pressure, Polish Academy of Sciences, Sokolowska 29/37, PL-01142, Warsaw, Poland

[3]Military University of Technology, Gen. Sylwestra Kaliskiego 2, PL-00908, Warsaw, Poland

[4]Gdansk University of Technology, G. Narutowicza 11/12, PL-80233, Gdansk, Poland

[5]Solaris Synchrotron NSRC, Jagiellonian University, Czerwone Maki 98, PL-30392, Krakow, Poland

[6]Institute of Experimental Physics, Faculty of Mathematics, Physics and Informatics, University of Gdansk, Wita Stwosza 57, PL-80308, Gdansk, Poland

Keywords: perovskites; synthesis; crystallographic structure; X-ray spectroscopy; Raman spectroscopy

Corresponding author (D. Wlodarczyk) email – wlodar@ifpan.edu.pl

TABLE OF CONTENTS:

3. RESULTS & DISCUSSION S3

3.1 Macro- and SEM microphotographs S3

Figure S1 – photograph of SCWO pellet superheated in air S3

3.4 Raman & FTIR Spectroscopies S4

Table S1 – group theory calculations for SCWO S4

3.5 Material stability at high temperatures S4

Figure S2 – DSC, TGA & Heat capacity data of SCWO in air and N_2 S5

4. Summary S7

Table S2 – summary of all standardized CIF data S7

Note: The section numbers here correspond to those in the main text, where the supporting figures and tables were referenced.

3. RESULTS AND DISCUSSION

3.1 Macro- and SEM microphotographs.

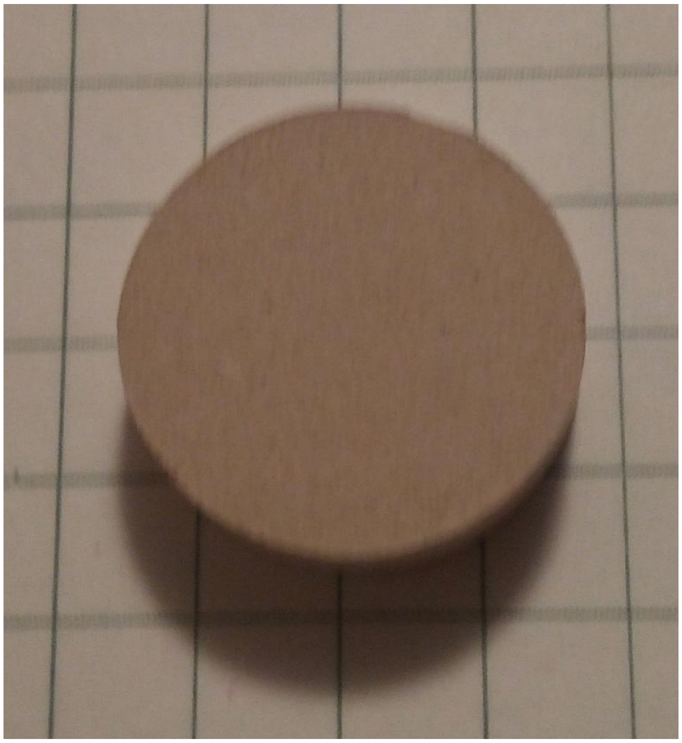

Figure S1. Photograph of SCWO heated in air up to 600°C. SCWO decomposes gradually into $Sr_3Ce_2W_2O_{12}$, Sr_2CeWO_6, and $SrWO_4$ mixed with CeO_{2-x}.

3.4. Raman & FTIR Spectroscopies.

Table S1. Group theory analysis for $Sr_9Ce_2W_4O_{24}$ (SCWO) tetragonal ($I4_1/a$) space group.

Material (SG)	Ion	Wyckoff Positions	Point Symmetry	Red. Representation
**SCWO**	A^{2+} (Sr)	8e, 16f	C_2, C_1	$4A_g+4A_u+4B_g+5^1E_g+5^1E_u+5^2E_g+5^2E_u$
	$B^{3/4+}$ (Ce)	8e, 16f	C_2, C_1	$4A_g+4A_u+4B_g+5^1E_g+5^1E_u+5^2E_g+5^2E_u$
I4₁/a	$B'^{6/5+}$ (W)	8c, 8d	C_i	$6A_u+6^1E_u+6^2E_u$
(C₄ₕ)	O^{2-}	16f	C_1	$3A_g+3A_u+3B_g+3^1E_g+3^1E_u+3^2E_g+3^2E_u$

$\Gamma_{TOTAL} = 11A_g+17A_u+11B_g+13^1E_g+19^1E_u+13^2E_g+19^2E_u$; $\Gamma_{ACOUSTIC} = A_u+{}^1E_u+{}^2E_u$;
$\Gamma_{IR} = 16A_u+18^1E_u+18^2E_u$; $\Gamma_{Raman} = 11A_g+11B_g+13^1E_g+13^2E_g$

3.5. Material stability at high temperatures

Differential scanning calorimetry (DSC) & Heat Capacity (C_p) measurements were performed using the Netzsch Phoenix DSC apparatus, model DSC 204 F1. Temperature scans were conducted from 300–873K at a heating rate of 10 K min^{-1}. A three-stage, heating–cooling–heating protocol was used under inert gas–inert gas–air conditions to assess the samples' behavior in both environments. Sapphire was used as a heat capacity reference. Crucibles were made of concave aluminum pans.

Thermogravimetry (TG) was carried out separately in air and nitrogen atmospheres (20 ml/min nitrogen flow) using a Netzsch Tarsus TG 209 F3 at temperatures ranging from 300 to 1273 K. The heating/cooling rate was approximately 10 K min^{-1}. Each sample, weighing approximately 10 mg, was placed in a small corundum crucible.

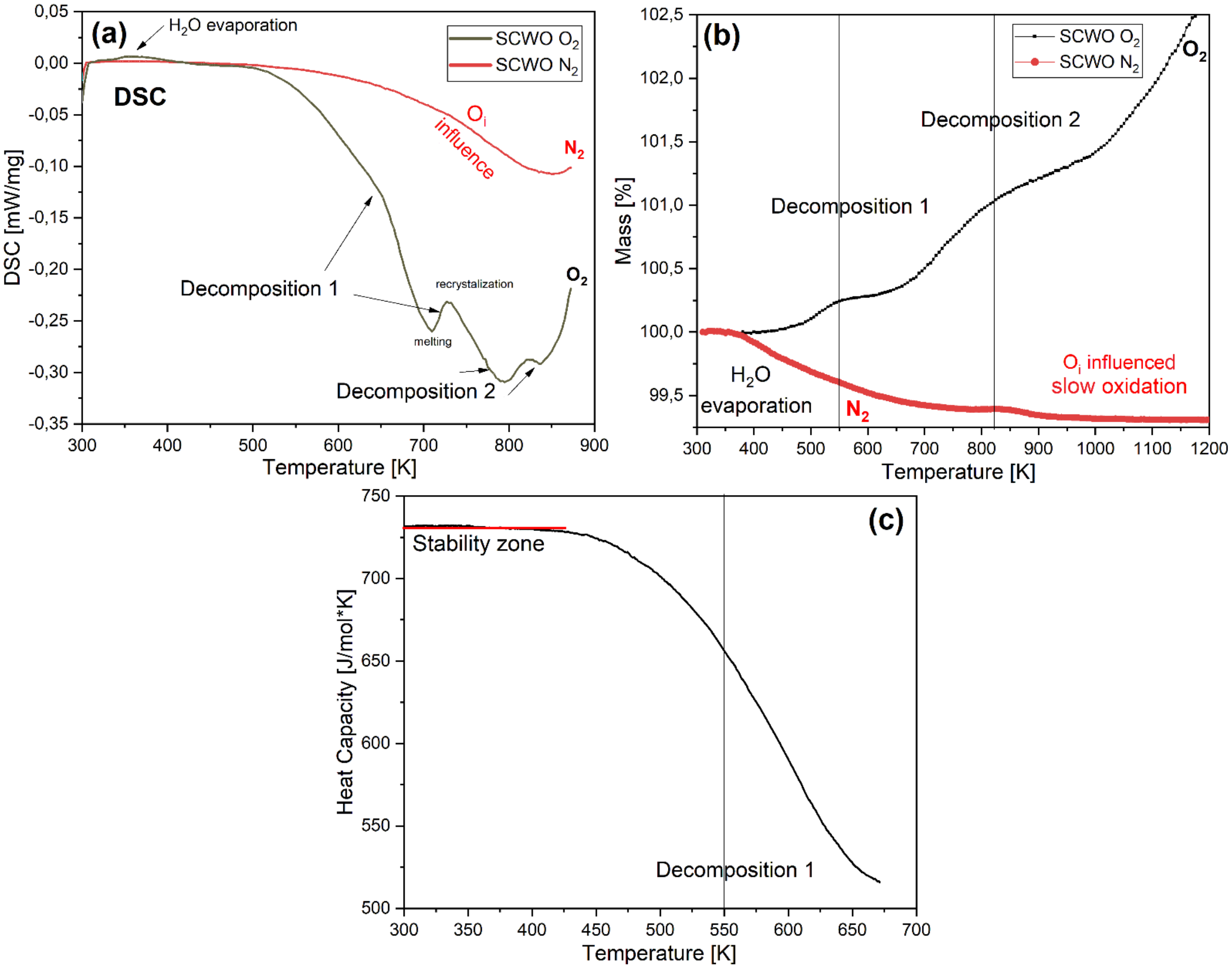

Figure S2. Differential scanning calorimetry (a) and thermogravimetry (b) measurements of $Sr_9Ce_2W_4O_{24}$ (SCWO) in the air (black) and in inert N_2 gas (red) at high temperatures, showing decomposition of the material in the former medium. (c) Heat capacity (C_p) measured against a sapphire reference. The average C_p was calculated in the air-stable region, indicated by the red horizontal line.

Complementary changes to those presented in Figure 10 of the main manuscript (featuring high temperature XRD & Raman results) occur in the same temperature ranges denoted as 'Decomposition 1' & 'Decomposition 2', as shown in the DSC & TG data in Figure S2.

In conjunction with information on minor phases (CeO_{2-x}, $Sr_3Ce_2W_2O_{12}$, $Sr_5W_2O_{12}$, Sr_2WO_5, $SrWO_4$) obtained from high-temperature XRD patterns, one can explain the reactions occurring during heating. The first reaction, occurring at temperatures up to about 400 K, is dehydration:

$Sr_9Ce_2W_4O_{24} \times n\ H_2O \rightarrow n\ H_2O\ (g) + Sr_9Ce_2W_4O_{24}$ accompanied by a slight loss of mass. Above ~550K, the situation changes drastically. In air, the calorimetry curve (a) shows a pronounced exothermic dip indicating oxidation, and in (b), a subsequent mass gain is observed. The heat capacity decreases sharply as the material decomposes into other, less complex components.

The first decomposition stage occurs at ~ 550 K and is related to Ce^{3+} oxidation:

$$Ce^{3+} \rightarrow Ce^{4+}\ ;\ 2\ Ce_2O_3 + O_2 \rightarrow 4\ CeO_2\ ;\ 14\ Ce_2O_3 + 3\ O_2 \rightarrow 4\ Ce_7O_{12}\ (CeO_{2-x});$$

$$6\ Ce_2O_3 + O_2 \rightarrow 4\ Ce_3O_5\ (CeO_{2-x})\ ;\ Sr_9Ce_2W_4O_{24} \rightarrow Sr_3Ce_2W_2O_{12} + 2\ Sr_2WO_5 + SrO$$

The second decomposition occurs at much higher temperatures (~750 K) and is mainly related to tungsten oxidation:

$$W^{5/6+} \rightarrow W^{6+}\ ;\ 2\ WO_{3-x} + x\ O_2 \rightarrow 2\ WO_3\ ;\ 2\ W_{18}O_{49}\ (W^{5/6+}O_{3-x}) + 5\ O_2 \rightarrow 36\ W^{6+}O_3$$

$$2\ Sr_3Ce_2W_2O_{12} + O_2 \rightarrow 4\ CeO_2 + 2\ Sr_2WO_5 + 2\ SrWO_4$$

After the second decomposition, the amount of some products increases over time due to the increasing free energy and spontaneous breaking of activation barriers:

$$2\ SrO + 4\ Sr_2WO_5 + O_2 \rightarrow 2\ Sr_5W_2O_{12}$$

It is worth noting that, even in inert N_2 gas, some changes still occur within SCWO. There is still a slight dip in the red curve in Figure S2(a), although without any pronounced features. In the TG curve (b), the mass also slightly increases. This may be related to the gradual oxidation of Ce and W ions via reaction with trapped interstitial oxygen. In the temperature range up to 450 K, (Figure S2(c)), the heat capacity (C_p) remains stable because the matrix undergoes no significant chemical changes. Therefore, the average molar C_p of SCWO was assessed solely for this stable region in the air: ~730.9 ± 0.9 J/(mol×K); median: ~731.0 J/(mol×K).

4. Summary

Table S2. Standardized CIF data published in the CCDC database determined from a Rietveld-refined powder XRD diffractogram of $Sr_9Ce_2W_4O_{24}$ hosting the tetragonal $I4_1/a$ space group.

**Chemical formula**		$Sr_9Ce_2W_4O_{24}$	
**Analytical formula**		$Sr_{9.22}Ce_2W_{3.82}O_{25.2}$	
**Formula weight [g/mol]**		2189.52	
**Melting Point [K]**		1743	
**Source**		X-ray laboratory	
**Temperature [K]**		298	
**Pressure [atm]**		1	
**Wavelength [Å]**		1.5406 (Cu $K\alpha_1$)	
**Crystal System**		Tetragonal	
**SG No.**		88	
**Dimensions**	a=b	c	α=β=γ
**Size [Å]**	11.6220(9)	16.2980(1)	90°
**V [$Å^3$]**		2201.421	
**Z**		4	
**d [g/cm^3]**		6.602	
**χ2**		4.51	
**R_p**		11.1	
**R_{wp}**		13.2	
**R_B**		3.81	
**R_{EXP}**		6.24	
**GoF**		223.05	
